# Protein Structure–Function Relationship: A Kernel-PCA Approach for Reaction Coordinate Identification

**DOI:** 10.1021/acs.jctc.5c00483

**Published:** 2025-07-14

**Authors:** Parisa Mollaei, Amir Barati Farimani

**Affiliations:** † Department of Mechanical Engineering, 6612 Carnegie Mellon University, Pittsburgh, Pennsylvania 15213, United States; ‡ Department of Biomedical Engineering, 6612 Carnegie Mellon University, Pittsburgh, Pennsylvania 15213, United States; ¶ Machine Learning Department, 6612 Carnegie Mellon University, Pittsburgh, Pennsylvania 15213, United States

## Abstract

In this study, we propose a Kernel-PCA model designed to capture structure–function relationships in a protein. This model also enables the ranking of reaction coordinates according to their impact on protein properties. By leveraging machine learning techniques, including Kernel and principal component analysis (PCA), our model uncovers meaningful patterns in the high-dimensional protein data obtained from molecular dynamics (MD) simulations. The effectiveness of our model in accurately identifying reaction coordinates has been demonstrated through its application to a G protein-coupled receptor. Furthermore, this model utilizes a residue-level dynamical network approach to uncover correlations in the structural dynamics of residues that are strongly associated with a specific protein property. These findings underscore the potential of our model as a powerful tool for protein structure–function analysis and visualization.

## Graphical abstract

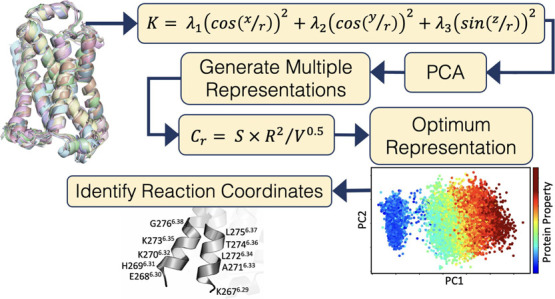

## 1 Introduction

Proteins serve as fundamental components of living organisms.^1–3^ The three-dimensional atomic arrangement determines the structure, which in turn dictates their function. They are dynamic entities that undergo conformational changes in response to various stimuli or interactions with other molecules. It often leads to biological functions such as substance release or enzymatic activity.^4–6^ Since structural changes directly influence biological function, deciphering structure–function relationships in proteins has long been a central pursuit in molecular biology. This endeavor is crucial to understanding the molecular mechanisms behind essential biological functions and diseases.

To achieve this understanding, extensive structural data is required. Experimental biology methods typically provide limited structural data for proteins, usually only their stable conformations, such as folded and unfolded states. On the other hand, molecular dynamics (MD) simulations^7,8^ have the capability to generate thousands of diverse protein structures, covering all possible states. Determining structure–function relationships using MD simulation trajectories is also challenging due to their high dimensionality in space and sequential time dependence. A promising solution to this challenge is efficient data reduction achieved through optimal protein representation. Protein representation refers to the way proteins are modeled and described in terms of their critical features.

This representation is capable of identifying functional sites, interaction networks, dynamic behavior, and conformational changes in biological processes. In the field of drug discovery,^9–11^ precise protein representation facilitates the discovery of potential drug targets and predicting the outcomes of molecular interactions. The representation is also vital in protein engineering,^12–14^ where the design of novel proteins with desired functions relies on a deep understanding of their structure–function relationships. In addition, the representation will aid in the modeling and simulation of complex protein systems.^15^ Such needs underscore the importance of developing a representation that captures both static and dynamic features determining the protein properties. Moreover, they should effectively present the maximum variations in protein dynamics to minimize the data required for exploring structure–function relationships. In this study, we introduce a model aimed at creating such a representation while addressing critical questions, including the following: Is this model generalizable to all proteins with different sequences and structures when utilizing raw atomic coordinates? Is it feasible to use only a subset of atoms to obtain a reduced representation? How could we reveal the reaction coordinates by using this representation? Is the model capable of identifying the extent of contribution of individual residues to the overall protein properties? To answer these questions, we took advantage of machine learning (ML) methods, as they offer computational tools and methodologies essential to dealing with high-dimensional protein data.^16–26^

The two well-known techniques for feature extraction and dimensionality reduction are Kernels^27,28^ and principal component analysis (PCA),^29,30^ respectively. Kernels offer a flexible framework for projecting data sets into higher-dimensional spaces. This enables the transformation of protein data into feature map spaces that enhance learning. PCA is a widely used method to reduce the dimensionality of data, while preserving its essential features. It is a linear technique that captures the directions (principal components) in which the data vary the most. By integrating these two methods, we developed a model that effectively analyzes protein structure–function relationships using MD trajectories and provides a meaningful representation for it.

While techniques such as t-SNE (t-distributed Stochastic Neighbor Embedding)^31^ and UMAP (Uniform Manifold Approximation and Projection)^32^ are widely used for visualizing high-dimensional biological data, they present limitations when applied to the identification of reaction coordinates. Both methods are designed primarily for visualization and are noninvertible, meaning they do not provide a direct mapping back to the original feature space. This lack of invertibility hinders interpretability, making it difficult to trace contributions back to specific residues or atomic features. Additionally, they are highly sensitive to hyperparameters, such as perplexity in t-SNE or the number of neighbors in UMAP, which can significantly alter the output embeddings and reduce reproducibility. In contrast, Kernel-PCA offers a nonlinear transformation through kernel functions while retaining the eigenvalue-based structure of PCA, making it suitable for identifying and ranking reaction coordinates based on variance contribution. This structured framework facilitates more interpretable and consistent mapping between protein features and properties, aligning well with the goals of this study.

## 2 Method

Our analysis begins with the preparation of the protein trajectories, as outlined in detail in Supporting Information Section 1. Figure 1 illustrates the framework where raw atomic coordinates serve as input for a Kernel model (Section 2.1). Prior to calculating atomic angles, all protein structures were aligned to a common reference frame using backbone atoms, such as N, C, and Cα. The (*x*, *y*, *z*) coordinates were extracted from these aligned PDB files, and the origin refers to the coordinate system of the aligned structures, which is consistent across all frames. The Kernel outputs are then processed by PCA^29,30^ to extract the essential features. Later, the optimum representation is selected via a defined Correlation ratio (Section 2.2). Ultimately, this representation introduces reaction coordinates and highlights their extent of correlation with protein properties in sequential order (Section 2.3).

**Figure 1:**
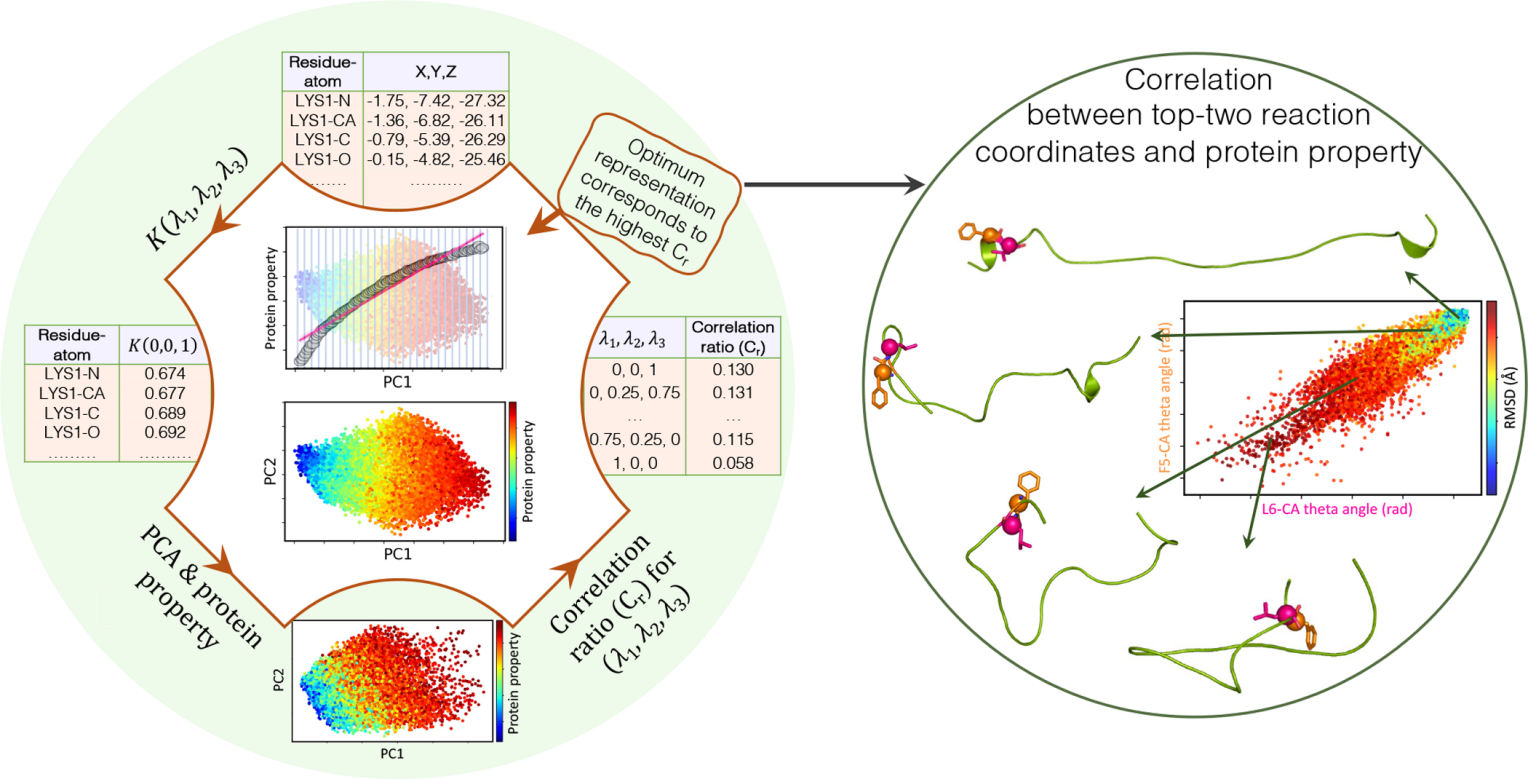
Overview of the Kernel-PCA model. Trajectories are initially prepared to provide atomic coordinates as input for a Kernel model (*K*(λ_1_, λ_2_, λ_3_)). It is followed by PCA to generate 2D representations for Kernel outputs. Finally, a defined Correlation ratio (*C*_r_) selects the optimal representation. This representation identifies and ranks reaction coordinates based on their significance to the protein property while also uncovering correlations among them.

### 2.1 Kernel Design for Effective Representation

Why is a Kernel model necessary? Initially, we opted to use PCA^29,30^ on raw data due to its ability to effectively identify principal components. These components are linear combinations of original features achieved by projecting high-dimensional data onto a 2D subspace. This subspace allows for efficient visualization and analysis. However, PCA alone may face challenges in extracting the intricate relationships within atom dynamics and their correlations to overall protein properties when the raw coordination of atoms is considered. This can be attributed to several factors, including the high dimensionality of the data, which can lead to high computational costs and overfitting. Moreover, some raw coordinate data may have less crucial structural information, making it challenging for PCA to effectively obtain maximum variations. Additionally, the presence of potential noise in the raw coordinates can further hinder the performance of PCA. Furthermore, PCA is a linear model, whereas complex coordinate data may exhibit nonlinear relationships. These challenges become particularly amplified when our goal is to generate a representation in which PC1 predominantly captures reaction coordinates and exhibits a strong relationship (roughly linear) with protein properties (Figure 2b,d). This choice of representation is motivated by its ability to significantly improve the identification of the reaction coordinates. To address these challenges, we initially developed a Kernel model.^27,28^

**Figure 2:**
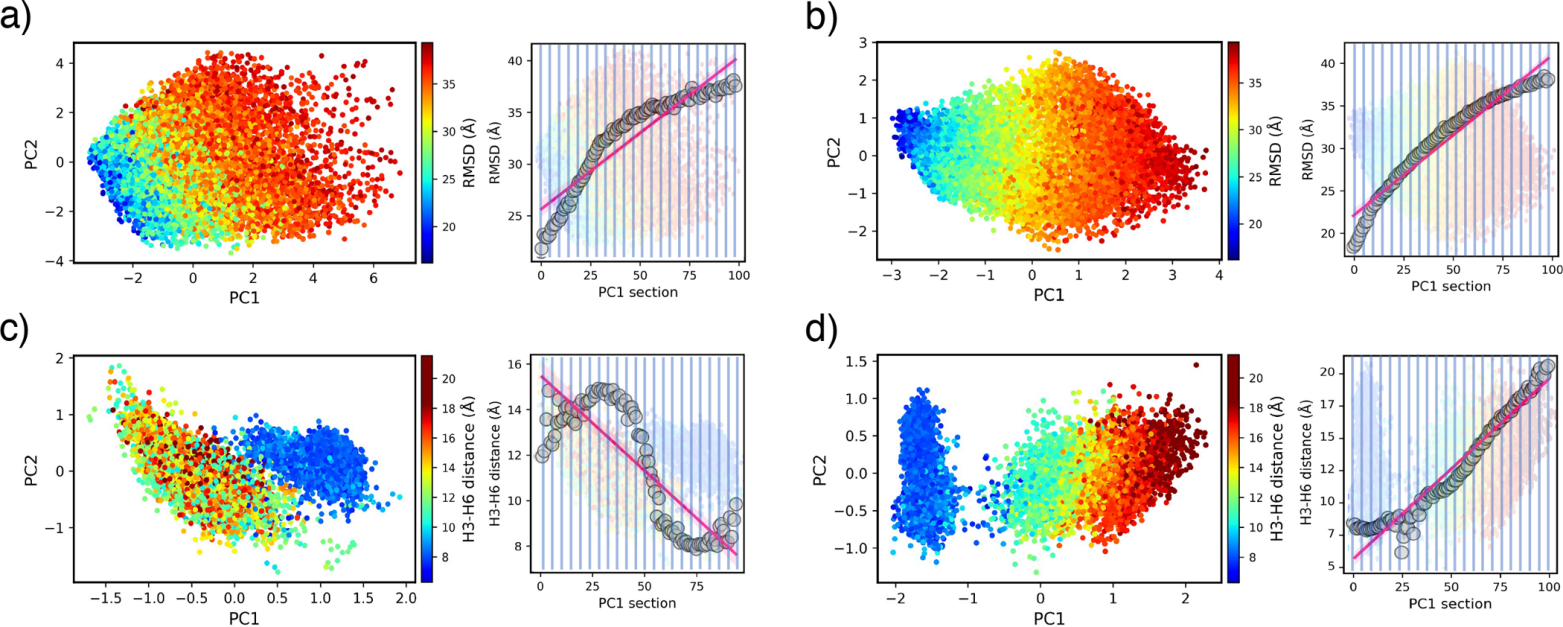
Kernel-PCA representations of the NTL9 protein (a,b) and β_2_ adrenergic receptor (c,d) corresponding to the lowest (a,c) and highest (b,d) *C*_r_ values. For the NTL9 protein: (a) *K*(0, 1, 0) with *C*_r_ = 0.05, (b) *K*(0.5,0.5,0) with *C*_r_ = 0.15. For the β_2_ adrenergic receptor (c) *K*(0,1,0) with *C*_r_ = 0.03, (d) *K*(0.75,0,0.25) with *C*_r_ = 0.13.

In our previous work,^23^ we demonstrated that the angles formed between atoms within individual residues are particularly valuable for identifying switch residues, which exhibit transitions between angular states correlated with protein function. Building on this foundation, we developed an angular Kernel model that utilizes atomic coordinates to capture conformational changes in proteins. We aimed to transform these data into a feature map space to uncover the conformation-function relationships. Later, the PCA effectively reduces the dimensionality of the transformed space to 2D while retaining crucial information. The Kernel model is defined as follows:

$$K=\lambda_{1}{\left(\cos\left(\frac{x}{r}\right)\right)}^{2}+\lambda_{2}{\left(\cos\left(\frac{y}{r}\right)\right)}^{2}+\lambda_{3}{\left(\sin\left(\frac{z}{r}\right)\right)}^{2}$$

where (*x*, *y*, *z*) represent the coordinates of individual atoms, and r denotes their Euclidean distance from the origin. The value of r is used to normalize the spatial coordinates when computing angular features in the Kernel transformation. (λ_1_, λ_2_, λ_3_) are hyperparameters. We established two constraints to limit the possibilities for hyperparameters and narrow the search space. We ensured that each λ_*i*_ fell within the range [0, 1] and the sum of the coefficients equaled 1. In this study, we selected five values for λ_*i*_ as [0, 0.25, 0.5, 0.75, 1], resulting in 15 combinations for (λ_1_, λ_2_, λ_3_) that confirm the summation equals 1.

It is important to note that studies have shown that the Kernel model enhances PCA performance compared to relying solely on *x*, *y*, or *z* coordinates (Supporting Information Section 2).

Figure 2 illustrates the impact of λ_*i*_ values in representations of the NTL9 protein and β_2_ adrenergic receptor. For both cases, *K*(0,1,0) generates representations where neither PC1 nor PC2 exhibit specific correlation with the proteins properties (Figure 2a,c), whereas this correlation is notably stronger with PC1 alone for *K*(0.5,0.5,0) and *K*(0.75,0,0.25) in the NTL9 protein and β_2_ adrenergic receptor, respectively (Figure 2b,d). After determining the optimal representation, we elaborate on how to identify reaction coordinates using this correlation between the protein properties and PC1.

While Figure 2b,d demonstrates the effectiveness of our Kernel-PCA representation, a quantitative comparison with t-SNE (Supporting Information, Section 3) shows that PCA yields a higher correlation ratio and is better suited for reaction coordinate identification due to its interpretability and reproducibility.

### 2.2 Evaluation of Representations Using the Correlation Ratio

Among different combinations of λ_*i*_ values, one of them yields a representation that provides the highest correlation between the PC1 and protein properties. To automate the identification of that one, we have established a metric for evaluating the Kernel’s performance based on the correlation between PC1 and protein property. We first divide the PC1 space into equal sections and calculate the average value of the protein property for 20 percents of datapoints in each section. We then fit the best linear curve on the average values to find its slope (*S*) and the coefficient of determination (*R* ^2^). In addition, we measured the average of total variances of the protein property within individual sections (*V*). The Correlation ratio (*C*_r_) is defined as follows:

$$C_{r}=\frac{S\times R^{2}}{V^{0.5}}$$

where the highest *C*_r_ introduces the optimal representation with the strongest correlation between protein properties and PC1. We assessed the impact of 15 combinations of λ_1_, λ_2_, and λ_3_ on correlation ratios for Protein B, NTL9, Trp-Cage, and Chignolin. For details, see Supporting Information Section 4.

Generating the optimal representation involved using all atomic coordinates within each structure (for example, the β_2_ adrenergic receptor used in this study contains 2175 atoms) across thousands of trajectories. However, this approach will lead to high computational costs for large proteins. We experimented with using only a single type of atom to develop a reduced representation. Initially, we considered individual atom types (such as O, N, C, CA, etc.) and used each one’s coordinates for the Kernel-PCA model. Ultimately, CB atoms exhibit the highest *C*_r_ value compared with other atoms. We observed that in large proteins like the β_2_ adrenergic receptor, using only CB atoms yields a higher *C*_r_ value compared to using all atom types. This improvement may stem from filtering out noise, specifically less important atomic information, from the input data.

To evaluate the effectiveness of CB representation versus all-atoms representation, we defined *R*_CB,T_ as the ratio of *C*_r_ for only CB atoms to *C*_r_ for total atoms. The *R*_CB,T_ value for the β_2_ adrenergic receptor is 111.27%. For Protein B, NTL9, Trp-Cage, and Chignolin *R*_CB,T_ values are 99.64, 89.23, 93.01, and 87.84%, respectively. *R*_CB,T_ values indicate that both using all atoms and using only CB atoms yield mostly similar results for representation as well as reaction coordinates. This approach led to a significant reduction in the feature space. In the case of the β_2_ adrenergic receptor, it decreased from 2175 (total atoms) to 260 (CB atoms) in a single structure. This reduction simplifies the model complexity, improves computational efficiency, and focuses on more relevant features, potentially enhancing the Kernel-PCA model performance.

### 2.3 Reaction Coordinate Identification

The aim of generating the optimum representation is to obtain the ranked reaction coordinates. These coordinates serve as essential variables that distill the complex protein system into essential variables that govern the protein’s function and biochemical processes. In this study, the ranked reaction coordinates refer to the structural features contributing to the protein’s properties, arranged according to their level of association. For the reaction coordinate analysis, we focused on the dynamics of individual amino acids in proteins. Since the dynamic of the α-carbon atom (CA) is sufficient to learn the dynamics of the entire amino acid,^33^ we measured theta angles (arccos­(*z*/*r*)) of the atom in every single residue over proteins. Each residue’s theta angles were then projected onto the optimum representation for *C*_r_ calculation. Since the highest *C*_r_ value for the protein property identified the optimal representation, the *C*_r_ values of individual features over this representation effectively measure their contribution to the property. As a result, we ranked the theta angles (reaction coordinates) based on their *C*_r_ values. The top-ranked ones reflect the most dominant modes of motion at the residue level, driving the protein’s property.

## 3 Data Set and MD Simulation

To assess the effectiveness and generalizability of our model, we benchmarked it using one large protein and four small proteins. For the large one, we selected the β_2_ adrenergic receptor, which consists of seven transmembrane helices (PDB: 2RH1). The original MD simulation data set^34^ was performed with the partial inverse agonist carazolol. The receptor is embedded within a POPC lipid bilayer using the AMBER03 force field for proteins, water, and ions, and the Berger united atom force field for lipids. Ligands were parametrized with the GAFF force field. The systems consisted of 58,406 to 59,044 atoms and were solvated with TIP3P water molecules with 0.15 M NaCl.^35^ Energy minimization was performed using steepest descent, followed by 100 ps of NVT equilibration (v-rescale thermostat, 300 K, τ = 0.1 ps) and 100 ps of NPT equilibration (Nose–Hoover thermostat, 300 K, τ = 0.2 ps; Parrinello–Rahman barostat, 1 bar, τ = 5 ps). All simulations used a 2 fs time step and periodic boundary conditions. The data analyzed in this work were aggregated from thousands of short trajectories totaling hundreds of microseconds in cumulative simulation time. The simulations were executed in parallel on Google’s Exacycle platform^36^ and performed using the Gromacs 4.5.3 MD package.^37^ Using these simulations, we can explore the activation process correlated with conformational changes in the receptor. For this protein, we randomly sampled 10,000 structures within 5000 MD simulations.

For the small proteins, we used all-atom MD simulation trajectories for Protein B (PDB: 1PRB), NTL9 (PDB: 2HBA), Trp-Cage (PDB: 2JOF), and chignolin (PDB: 5AWL).^38–40^ These simulations investigated the folding process in these proteins with diverse structures ranging in length from 10 to 47 amino acids. Protein B and Trp-Cage contain α helices, while Chignolin stands out as a purely β-sheet protein, and NTL9 displays mixed αβ structures. To generate the original data set, MD simulations were initiated from random coil conformations for each protein. They involved solvating all initial structures in cubic boxes, ionizing them according to the Lindorff-Larsen et al.^40^ protocol, and employing ACEMD^41^ on the GPUGRID.net network.^42^ The CHARMM22*^43^ force field and TIP3P water model^35^ were utilized at a temperature of 350 K. For these proteins, we randomly sampled 10,000 structures from the original MD simulation trajectories.

## 4 Results

We aim to find optimum representations and capture the top-ranked reaction coordinates. The diverse set of proteins, varying in size and structure with different properties, allows us to assess the generalizability of our model.

### 4.1 Case 1: Reaction Coordinates in the β_2_ Adrenergic Receptor

G protein-coupled receptors (GPCRs) are a diverse group of cell surface receptors that undergo conformational changes while interacting with ligands.^44–46^ Due to ligand binding, GPCRs become activated and trigger intracellular signaling cascades, leading to various physiological processes within the body.^47–49^ GPCRs consist of seven transmembrane helices (TMs), among which the key conformational change during receptor activation primarily involves the movement of transmembrane helix six (TM6).^50,51^ We analyzed the structure–function relationship in the β_2_ adrenergic receptor (β_2_AR).^52,53^ Figure 3a depicts the motion of TM6 between the inactive (blue: PDB 2RH1)^54^ and active (red: PDB 3P0G)^55^ structures of β_2_AR. Typically, this conformational change is identified by measuring the contact distance between TM6 and TM3.^34^ In this study, we measured the C_α_ contact distance between R131^3.50^-L272^6.34^ residues in the receptor (denoted as the H3–H6 distance). Figure 3a also highlights H3–H6 distance increases from 8.4 to 14.1 Å in the transitions from inactive to active states. Given the size of this receptor, we utilized only the CB atoms when generating representations (see Section 2.2). Due to the strong correlation between H3–H6 distances and activation states, we established the optimal representation based on these distances. Figure 3b illustrates that the chosen representation offers a strong correlation of receptor activation with PC1. Therefore, we can analyze reaction coordinates within this representation, as discussed in Section 2.3.

**Figure 3:**
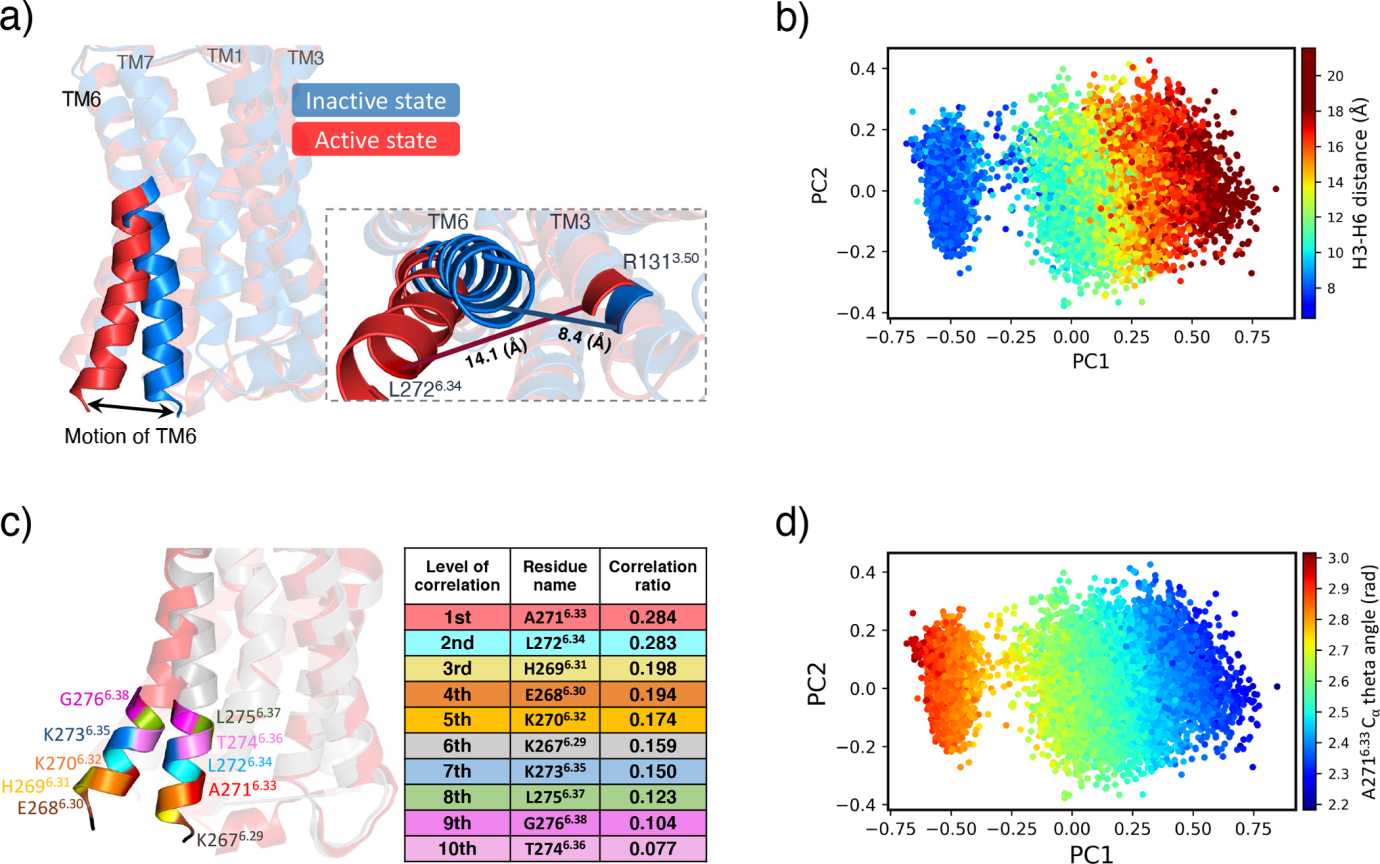
Identification of reaction coordinates in β_2_AR using the Kernel-PCA model. (a) Known reaction coordinates in β_2_AR are the intracellular parts of TM6 during the transition from the inactive to active state. (b) H3–H6 distances mapped onto the optimum representation. (c) Our model identifies the top ten reaction coordinates located in the intracellular parts of TM6 (aligns with part (a)), where essential conformational changes occur during activation process. (d) Dynamic motion of A271^6.33^ projected onto the optimum representation.

Figure 3c displays the top ten reaction coordinates in the receptor’s conformation that are marked with colors, corresponding to those listed in the table. Notably, all of them are located in the intracellular part of TM6, where the receptor undergoes the most significant motion during the activation process. This attests to the strength of our model in capturing the essential structural features correlated to the protein’s property.

Figure 3d demonstrates the dynamic motion of A271^6.33^, as the first-ranked reaction coordinate, mapped on the selected representation. Comparing it with Figure 3b, we observe that the theta angle in the A271^6.33^ residue decreases when the protein becomes activated. Such relationships can be identified by comparing the pattern of distribution of each feature across the representation with its corresponding pattern in the property. For further investigation, the representations of reaction coordinates listed in Figure 3c are illustrated in Supporting Information Section 5 and Figure S5.

### 4.2 Case 2: Reaction Coordinates in Protein B, NTL9, Trp-Cage, and Chignolin

We assessed the folding states of Protein B (PDB: 1PRB), NTL9 (PDB: 2HBA), Trp-Cage (PDB: 2JOF), and Chignolin (PDB: 5AWL) proteins by measuring the Root Mean Squared Displacement (RMSD)^56^ relative to their nonfolded reference structures (see Supporting Information Section 1). For these proteins, Figure S4 displays normalized *C*_r_ values plotted against λ_*i*_ values, illustrating how different combinations of λ_*i*_ affect the *C*_r_ value. Moreover, Table S2 lists the λ_*i*_ values corresponding to the highest and lowest *C*_r_ values.

For the small proteins, Figure 4 depicts the optimum representations generated using all atoms as well as only the CB atoms, colored by RMSD values. The figures are followed by the corresponding fitted lines that maximize *C*_r_ values. Table 1 lists the top three reaction coordinates in each protein, introducing the key residues correlated with folding states.

**Table 1:** Top Three Most Significant Reaction Coordinates in Protein B, NTL9, Trp-Cage, and Chignolin, Introducing the Motion Dynamics of Residues with the Highest Contribution to the Overall RMSD

protein	first-ranked residue	second-ranked residue	third-ranked residue
protein B	N35	E33	V34
NTL9	L6	K7	F5
Trp-cage	Y4	P19	A3
chignolin	Y2	W9	P4

**Figure 4:**
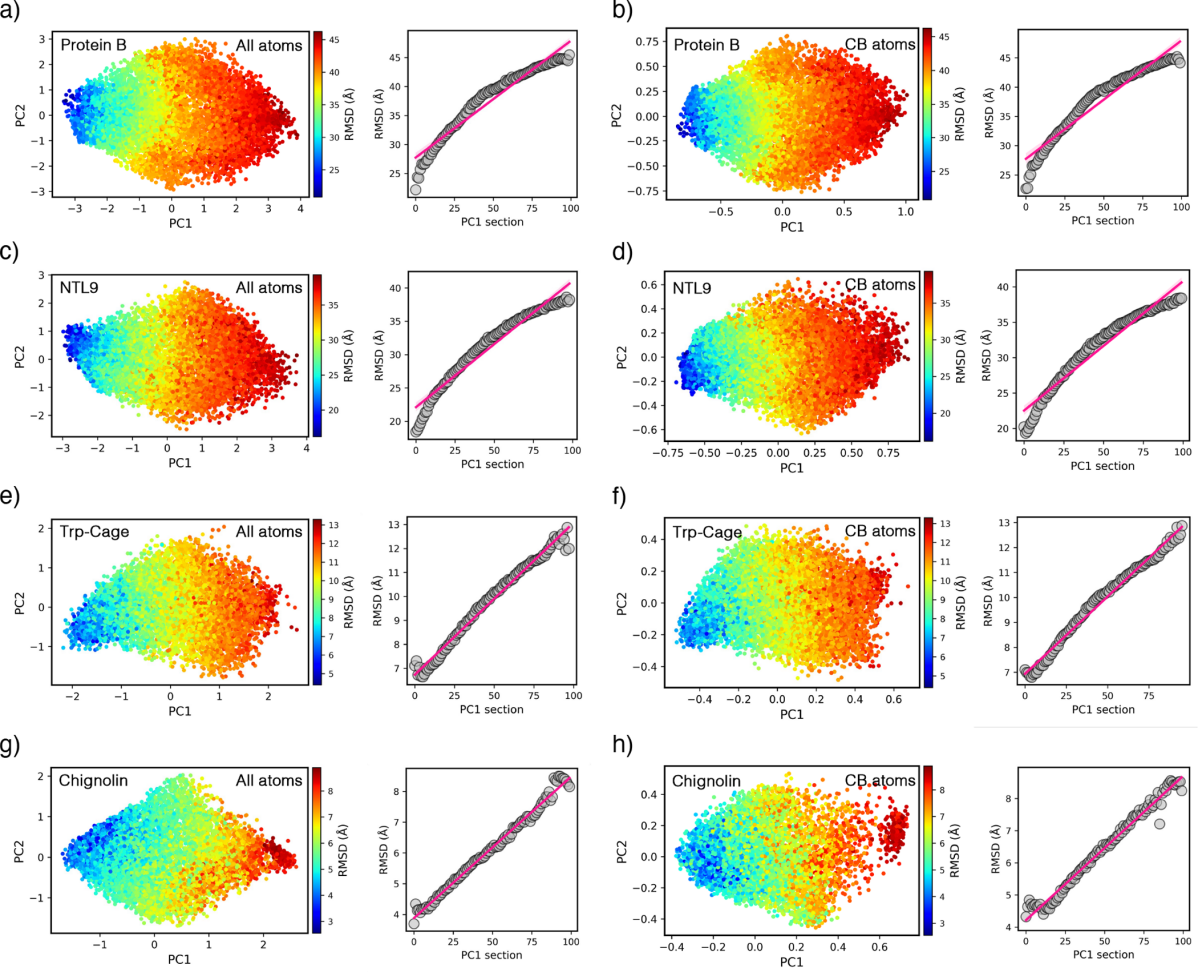
RMSDs projected onto the optimal representation, illustrating the maximum correlation ratio achieved using all atoms for (a) protein B, (c) NTL9, (e) Trp-cage, and (g) chignolin. Panels (b,d,f,h) show their corresponding representations generated using only the CB atoms.

In Trp-Cage, P19 residue is part of the hydrophobic core that stabilizes the folded structure.^57,58^ Their side chains interact to form a hydrophobic staple that is critical for the stability of the protein. In Chignolin, residues Y2 and W9 form a hydrophobic cluster that stabilizes the native β-hairpin fold.^59^ The folding process begins with turn formation, where P4 is positioned, followed by hydrophobic packing between Y2 and W9. These residues thus play distinct yet complementary roles in guiding the protein into its folded conformation.^59^

More studies are required to understand the role of N35, L6, Y4, and Y2 as the first-ranked reaction coordinates in Protein B, NTL9, Trp-Cage, and Chignolin in their folding mechanisms. Extending this list to include all residues as the ranked reaction coordinates allows us to analyze the internal interactions of each residue, which demonstrate their collective impact in harmony.

## 5 Network-Based Insights into Reaction Coordinate Relationships Linked to Protein Properties

We delved deeper to establish connections among the dynamical behaviors of reaction coordinates linked to protein properties (Figure 5). Our findings reveal that pairs of top-ranked reaction coordinates form a harmonious linear or diagonal pattern when they are strongly associated with the protein’s function. For example, in β_2_AR, the theta angles of residues A271^6.33^ and L272^6.34^ decrease simultaneously as the receptor becomes activated (Figure 5a). A similar pattern is observed in residues L6 and K7 of the NTL9 protein during folding, where both angles decrease as the protein approaches its native state (Figure 5b). See Supporting Information Section 6 for comparable correlations among the top-ranked reaction coordinates in Protein B, Trp-Cage, and Chignolin. These correlated behaviors can be interpreted as forming a residue-level dynamical network, where nodes represent residues and edges reflect strong covariation in their contributions to functionally relevant reaction coordinates. This network is not based on spatial proximity but rather on the temporal coordination of structural features that collectively influence the protein’s function. For example, in GPCRs, such networks reveal functionally coupled residues involved in activation. This network-based perspective provides insight into how groups of residues act in concert and can guide therapeutic strategies by identifying functionally critical interaction centers that mediate protein behavior.

**Figure 5:**
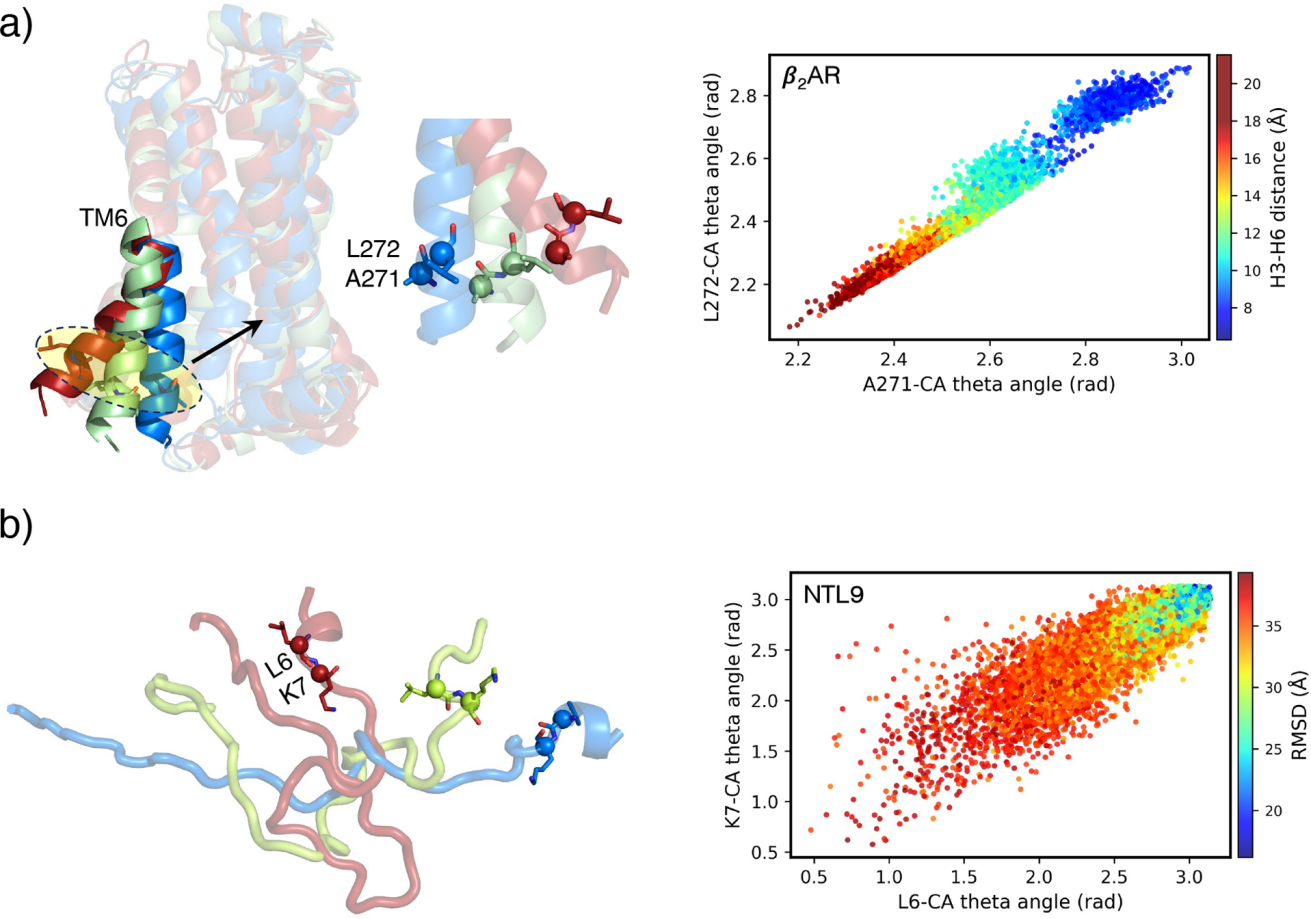
Strong correlation between the top two residues associated with the protein property. (a) Relationship between A271^6.33^ and L272^6.34^ residues in the inactive (blue), intermediate (green), and active (red) states of β_2_AR. (b) Interaction between L6 and K7 residues in the unfolded (blue), intermediate (green), and folded (red) states in NTL9 protein.

## 6 Conclusions

In this study, we introduced a Kernel-PCA model for efficient protein representation, aiming to elucidate structure–function relationships by identifying the reaction coordinates. These reaction coordinates are then ranked on the basis of their degree of contribution to the protein property. The flexibility of the representation, along with tunable hyperparameters, ensures applicability to various protein dynamics obtained through MD simulations. We initially evaluated the efficacy of our model by applying it to the β_2_ adrenergic receptor, a G protein-coupled receptor with a known structure-activation relationship. Subsequently, we applied the model to Protein B, NTL9, Trp-Cage, and Chignolin with unknown relationships between structural features and folding processes. Using this model, we can also establish interconnections among the dynamics of all residues linked to the protein properties through a network-based approach. This method provides a robust tool for understanding complex biological systems and advancing biotechnology applications.

## Supplementary Material



## Data Availability

The necessary information containing the codes and data for downstream tasks used in this study is available here: https://github.com/pmollaei/ProtKernel.

## Notes

Data preprocessing, protein representation using atomic coordinates, Kernel-PCA vs Kernel-t-SNE, correlation ratios correspond to *K*(λ_1_, λ_2_, λ_3_), top reaction coordinates in β_2_ adrenergic receptor, and relationships between top reaction coordinates in protein B, Trp-cage, and chignolin proteins (PDF)
