# Supplementary material for: Protein Structure–Function Relationship: A Kernel-PCA Approach for Reaction Coordinate Identification
Source: J Chem Theory Comput. 2025 Jul 14;21(14):7122–30. doi: 10.1021/acs.jctc.5c00483 (PMC12288012; doi:10.1021/acs.jctc.5c00483)
Supplement: Supplementary file 1 [file ct5c00483_si_001.pdf]

Supporting Information: Protein Structure-Function Relationship: A Kernel-PCA Approach for Reaction Coordinate Identification

Parisa Mollaei[†] and Amir Barati Farimani[*,†,‡,¶]

†*Department of Mechanical Engineering, Carnegie Mellon University, Pittsburgh, PA 15213, USA*

‡*Department of Biomedical Engineering, Carnegie Mellon University, Pittsburgh, PA 15213, USA*

¶*Machine Learning Department, Carnegie Mellon University, Pittsburgh, PA 15213, USA*

E-mail: barati@cmu.edu

1: Data Preprocessing

I. Reference protein structure:

To simulate protein dynamics, water and/or ions may be added to the protein's environment. To isolate only the protein structure, the surrounding environment must be purified. For the unfolded structure of small proteins, a chain of residues can also be constructed using molecular visualization tools such as Visual Molecular Dynamics (VMD).[1] In our study, we built protein B, NTL9, Trp-Cage and Chignolin through VMD and chose the purified inactive structure of the β_2 adrenergic receptor (PDB 2RH1)[2] as the reference structures.

To build the protein structure, follow these steps:

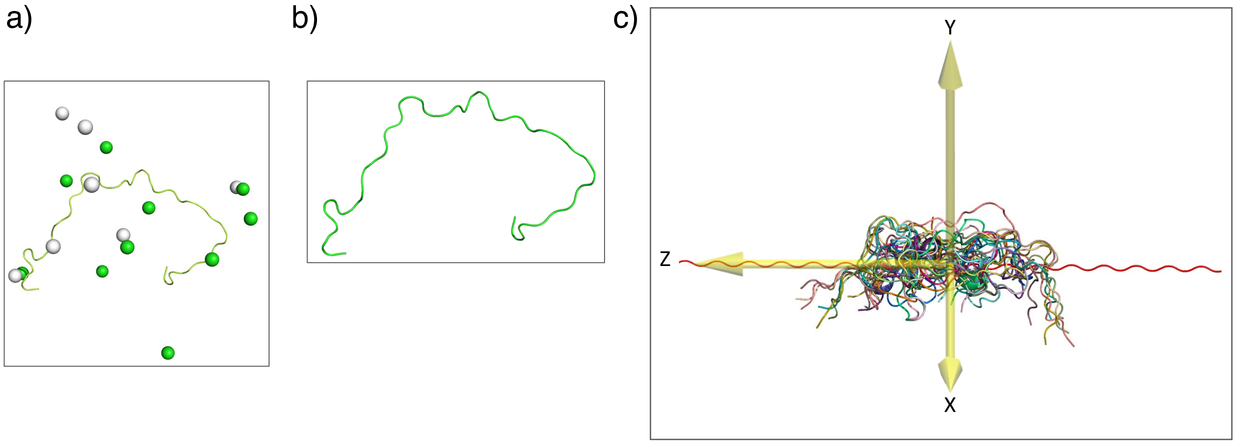

Figure S1: (a) Original protein structure with additional elements from MD simulations, (b) isolated protein structure after removing extra elements, and (c) aligned trajectory structures with respect to the oriented reference protein.

1. From components of a simulation (Figure S1a), select only the protein sequence (Figure S1b).

2. Build the reference protein structure using VMD (Main →Extensions →Modeling →Molefacture →Build →Protein Builder →Add the sequence from part 1→Select Straight →Build)

3. Save the PDB file of the reference protein. Later, you will align trajectory structures to this one (Figure S1c).

4. (Optional): Open the PDB file in text format. If the order of atoms in the generated PDB file does not match that of the trajectories, or if the residue indices are not sequentially ordered (e.g., the N atom of residue #2 is located among the atoms of residue #1), open the PDB file in PyMOL[3] and save it. Go to PyMOL →File →Export Molecule (choose pdb format). Later, repeat this process for the trajectories.

II. Optional: Draw a sphere at the origin and shift the geometric center of the reference protein to it

1. Load the reference protein in VMD. Use the "move_to_origin.tcl" command (see GitHub)

in the TKConsole or specify it in the VMD startup command.

III. Align principal axes of the reference protein with the x, y and z axes.

1. Download orient.tar.gz (here) and la101psx.tar.gz (here). The latter is a linear algebra package by Hume Integration Software; more information can be found on this website: http://www.hume.com/la/.

2. Unpack and load the packages and orient the protein using the "alignment_package.tcl" (see GitHub)

3. Save the oriented reference protein.

IV. Prepare simulation trajectories

1. If the simulation trajectories contain extra elements, save only the essential protein residues using the "Selected atoms" section in VMD (for example, resid 2 to 9). Ensure that the saved residues are compatible with the reference protein.

V. Align simulation trajectories to the reference protein

1. Load both the reference protein and the simulation trajectories into VMD. Ensure that both files display the same number of atoms on the VMD main screen.

2. Align the entire simulation trajectory with the reference protein. Extensions →Analysis →RMSD Trajectory Tools. Choose "Top" for the "reference mol" (ensure that the 'T' on the VMD main screen is associated with the reference protein) and select "Backbone" for the "Selection Modifiers".

3. Save the aligned simulation trajectories. They will be used as input for the Kernel model.

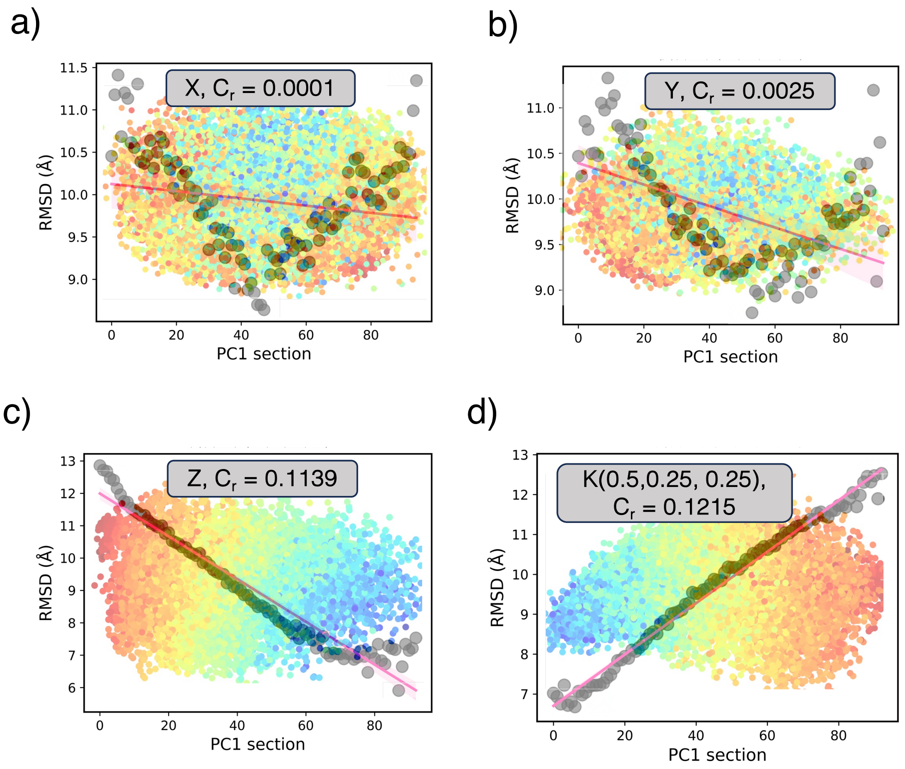

Figure S2: Protein representations of the Trp-Cage protein (PDB: 2JOF) obtained using dimensionality reduction techniques. (a–c) PCA applied separately to raw atomic coordinates in the (a) x, (b) y, and (c) z directions. (d) Optimal protein representation produced by the Kernel-PCA model, capturing non-linear structural relationships.

2: Protein Representation Using Atomic Coordinates

The preprocessed trajectories using only x, y and z coordinates of atoms may or may not yield an appropriate representation associated with the protein property, depending on the dynamic complexity of the protein. Figures S2 illustrate the RMSD projected onto the optimal representations generated solely from the x (a), y (b), and z (c) coordinates of atoms in the Trp-Cage protein (PDB: 2JOF). This analysis shows that, in terms of the Correlation ratio, the z-coordinate of atoms provides a better representation compared to the x and y coordinates. However, Figure S2d demonstrates that applying Kernel-PCA results in a representation with a higher Correlation ratio, offering a stronger association with RMSD. Thus, to achieve a generalizable representation that effectively captures key

reaction coordinates that influence global properties, we utilized the Kernel-PCA model.

3: Kernel-PCA vs Kernel-t-SNE

Table S1: Quantitative comparison of dimensionality reduction techniques (t-SNE and PCA) for the β_2-adrenergic receptor representation.

Method	Slope (S)	R^2	Avg. Variance (V)	Correlation Ratio (C_r)
PCA	0.13801	0.94413	0.94841	0.13380
t-SNE	0.12242	0.96521	1.21775	0.10708

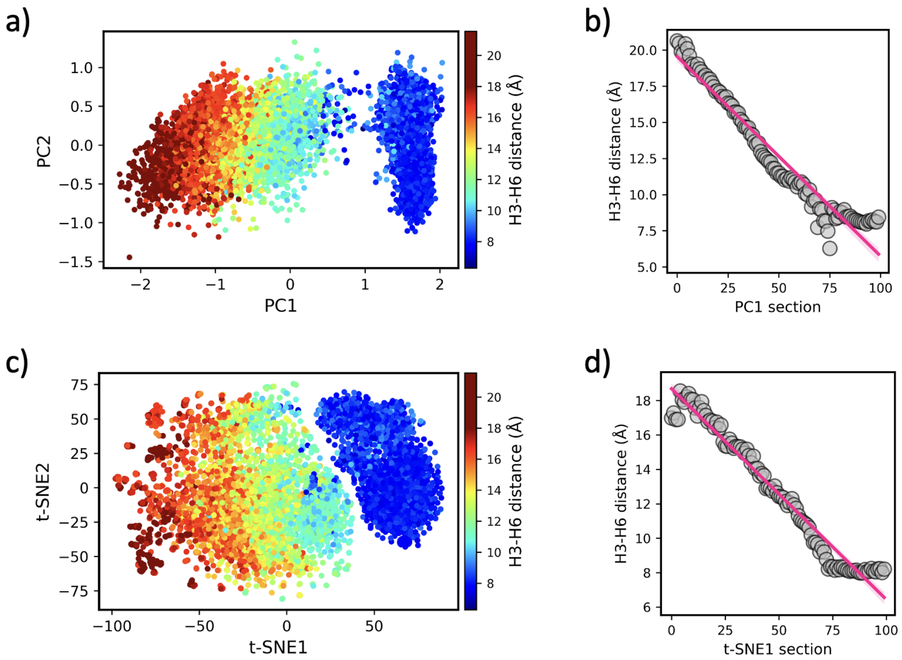

Figure S3: Comparison of PCA and t-SNE embeddings. (a, c) 2D projections colored by H3–H6 distance using PCA and t-SNE, respectively. (b, d) Correlation between the H3–H6 distance and PC1 or t-SNE1 section averages, fitted with linear regression to compute the Cr value. See Table S1 for corresponding variables.

We evaluated both PCA and t-SNE on the β_2 Adrenergic Receptor dataset to assess their effectiveness in identifying reaction coordinates (Figure S3). The comparison metrics include the slope (S) and R^2 from a linear fit of section-wise averages of H3–H6 distance, the average variance (V) within sections, and the derived Correlation Ratio (C_r). Results are shown in

Table S1. Although t-SNE showed a slightly higher R^2, its C_r value (0.10708) was lower than that of PCA (0.13380), indicating that PCA provides a higher accuracy in variance reduction. Additionally, t-SNE is non-invertible and highly sensitive to perplexity settings, limiting its interpretability for identifying reaction coordinates at the residue level. The PCA results correspond to the optimal Kernel-PCA representation with hyperparameters K(0.75, 0.0, 0.25), which is the same representation shown in Figure 2d of the main manuscript. This confirms that our Kernel-PCA model preserves functionally relevant motion and provides a robust, interpretable framework for identifying key residues in conformational transitions. Therefore, we excluded t-SNE from the main analysis and instead adopted PCA (and Kernel-PCA) to construct interpretable low-dimensional representations that can be directly associated with structural features and reaction coordinates.

4: Correlation Ratios Correspond to K(λ_1, λ_2, λ_3)

Table S2: The λ_1, λ_2, λ_3 values corresponding to the highest and lowest Correlation ratios.

Protein	Best ($\lambda_1, \lambda_2, \lambda_3$)	Worst ($\lambda_1, \lambda_2, \lambda_3$)
Protein B	(0.5, 0.5, 0)	(0, 1.0, 0)
NTL9	(0.5, 0.5, 0)	(0, 1.0, 0)
Trp-Cage	(0.5, 0.25, 0.25)	(1.0, 0, 0)
Chignolin	(0.25, 0, 0.75)	(1.0, 0, 0)

Table S2 reports the best and worst (λ_1, λ_2, λ_3) corresponding to the highest and lowest Correlation ratio. Figure S4 represents normalized Correlation ratios vs (λ_1, λ_2, λ_3) for Protein B, NTL9, Trp-Cage, and Chignolin. Although a specific combination (λ_1, λ_2, λ_3) could provide the highest Correlation ratio for each protein, the combination (0.5, 0.5, 0), highlighted on the figure, can generate the representation with a high Correlation ratio across different proteins.

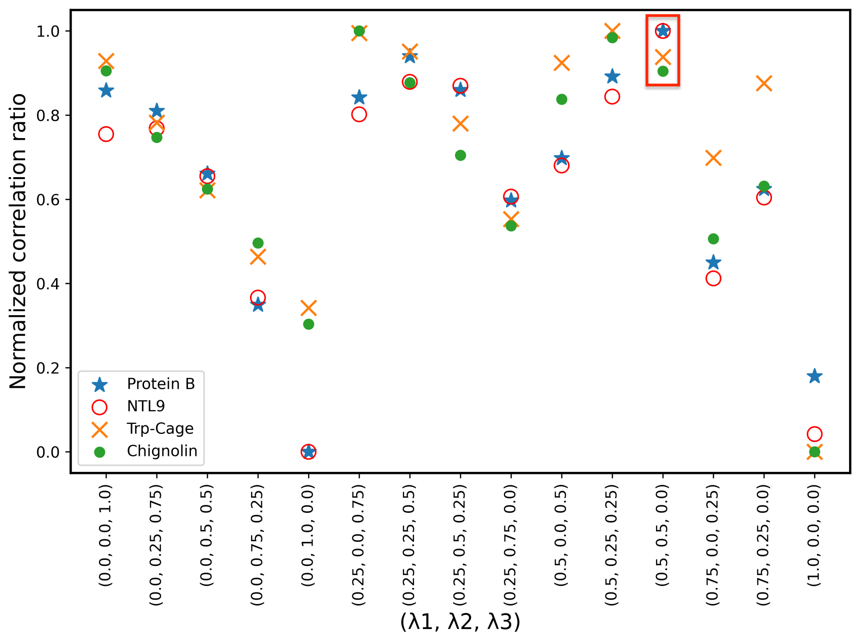

Figure S4: Normalized Correlation ratio obtained from different combinations of (λ_1, λ_2, λ_3) for Protein B, NTL9, Trp-Cage, and Chignolin.

5: Top Reaction Coordinates in β_2 Adrenergic Receptor

Figure S5 illustrates the top ten reaction coordinates in β_2AR, representing the theta angles of the CA atoms in the residues most correlated to the receptor activation process. As described in Figure 3 in the main manuscript, all the top ten residues are located in the intracellular part of TM6, where significant conformational changes occur during the activation process. Comparison of Figure S5 with Figure 3.b reveals a strong connection between dynamics of the top ten residues, both among themselves and with the activation state of the receptor.

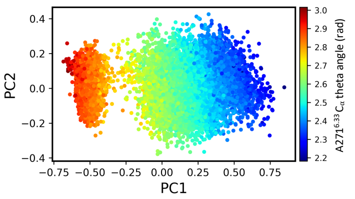

Level of correlation	Residue name	Correlation ratio
1st	A271$^{6.33}$	0.284
2nd	L272$^{6.34}$	0.283
3rd	H269$^{6.31}$	0.198
4th	E268$^{6.30}$	0.194
5th	K270$^{6.32}$	0.174
6th	K267$^{6.29}$	0.159
7th	K273$^{6.35}$	0.150
8th	L275$^{6.37}$	0.123
9th	G276$^{6.38}$	0.104
10th	T274$^{6.36}$	0.077

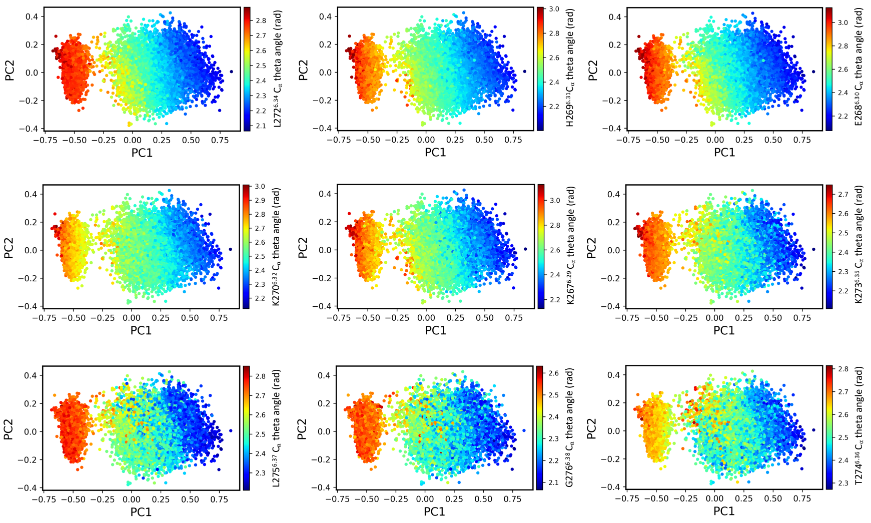

Figure S5: Top ten reaction coordinates of the β_2 Adrenergic Receptor, with each coordinate showing the dynamics of the theta angle in the CA atom of each residue, projected onto the optimal representation.

6: Relationships Between Top Reaction Coordinates in Protein B, Trp-Cage, and Chignolin Proteins

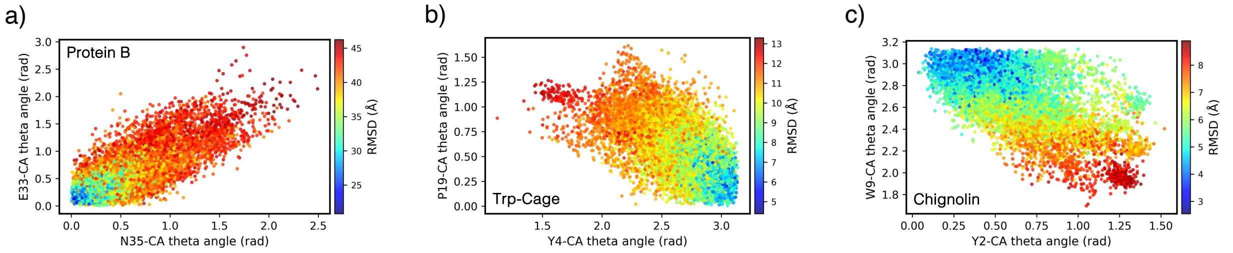

Figure S6: Top two reaction coordinates for (a) Protein B, (b) Trp-Cage, and (c) Chignolin proteins corresponding to the theta angles of CA atoms in the top two amino acids correlated with the folding state of each protein.

Figure S6 shows the relationships between the top two reaction coordinates corresponding to theta angles in two most associated residues with the folding states in protein B (a), Trp-Cage (b), and Chignolin (c) proteins. This information provides insight into the correlation between dynamics and conformations of these residues and their relationship with the overall protein property and function.

References

(1) Humphrey, W.; Dalke, A.; Schulten, K. VMD: visual molecular dynamics. *Journal of molecular graphics* **1996**, *14*, 33–38.

(2) Cherezov, V.; Rosenbaum, D. M.; Hanson, M. A.; Rasmussen, S. G.; Thian, F. S.; Kobilka, T. S.; Choi, H.-J.; Kuhn, P.; Weis, W. I.; Kobilka, B. K.; others High-resolution crystal structure of an engineered human β2-adrenergic G protein–coupled receptor. *science* **2007**, *318*, 1258–1265.

(3) DeLano, W. L.; others Pymol: An open-source molecular graphics tool. *CCP4 Newsl. Protein Crystallogr* **2002**, *40*, 82–92.
